# Prevalence and Predictors of Severity of Rebound Pain After Regional Anesthesia in Patients Undergoing Orthopedic Surgery: A Cross‐Sectional Study

**DOI:** 10.1155/prm/2130050

**Published:** 2025-12-05

**Authors:** Negesse Zurbachew Gobezie, Melaku Zewdu Yehualaw, Temesgen Birlie Asmare, Getachew Mekete Deress, Kumlachew Geta Belete, Diriba Teshome, Basazinew Chekol Demilew, Meraf Mehari Yitbarek, Kaletsidk Dessalegn Mossie, Begizew Yimenu Mekuriaw, Habtie Bantider Wubet

**Affiliations:** ^1^ Department of Anesthesia, School of Medicine, College of Health Sciences, Debre Tabor University, Debre Tabor, Ethiopia, dtu.edu.et; ^2^ Department of Midwifery, College of Health Sciences, Debre Tabor University, Debre Tabor, Ethiopia, dtu.edu.et

**Keywords:** orthopedic, rebound pain, regional anesthesia, severity, surgery

## Abstract

**Background:**

Rebound pain (RP) is an acute, burning, or dull‐aching type of pain that occurs following the resolution of regional anesthesia. It is a common but understudied problem in orthopedic patients undergoing surgery under regional anesthesia. This study aimed to determine the prevalence and predictors of RP severity in patients undergoing orthopedic surgeries.

**Methods:**

A cross‐sectional study was conducted involving 364 patients who underwent orthopedic surgery and received regional anesthesia. To determine factors associated with the severity of RP, bivariable and multivariable ordinal logistic regression analyses were conducted. Both crude and adjusted odds ratios with their corresponding 95% confidence interval were calculated to determine the strength of association.

**Result:**

The prevalence of RP after resolution of regional anesthesia was 70.9% (95% CI: 65.9–75.5). Among all participants, 30.2% experienced mild RP, 19.2% had moderate pain, and 21.4% reported severe RP. Age less than 60 years (AOR: 2.34, 95% CI: 1.38–3.95, and *p* :  0.001), ASA physical status III or above (AOR: 5.84, 95% CI: 2.17–15.69, and *p* < 0.001), and having moderate (AOR: 2.86, 95% CI: 1.21–6.78, and *p* : 0.017) and severe (AOR: 7.48, 95% CI: 4.09–13.68, and *p* < 0.001) preoperative pain were significantly associated with increased odds of RP. In contrast, premedication with intravenous dexamethasone (AOR: 0.24, 95% CI: 0.14–0.43, and *p* < 0.001) and using local anesthetic adjuvants (AOR: 0.05, 95% CI: 0.02–0.11, and *p* < 0.001) were significantly associated with reduced odds of severe RP.

**Conclusion:**

Our study found a high incidence of RP following the resolution of regional anesthesia in patients undergoing orthopedic surgery. To reduce its incidence and severity, it is essential to effectively manage and alleviate preoperative pain, incorporate local anesthetic adjuvants into nerve block solutions, utilize intravenous dexamethasone, and incorporate multimodal analgesia before the resolution of regional anesthesia.

## 1. Introduction

Orthopedic surgery is rapidly expanding globally, with continuous innovations and advancements aimed at managing musculoskeletal disorders, restoring mobility, and enhancing quality of life [1]. Due to the increasing prevalence of degenerative musculoskeletal conditions and road traffic accidents, the number of orthopedic procedures performed annually is increasing by approximately 4.9%. In 2017, approximately 22.3 million orthopedic surgical procedures were performed globally [2–4].

Patients undergoing orthopedic procedures are operated under general or regional anesthesia. In recent decades, since the introduction of ultrasound technology, the application of regional anesthesia techniques has evolved with anesthesia practice and become the technique of choice for various orthopedic procedures [5–7]. Regional anesthesia, such as peripheral nerve block, can be used as a sole surgical anesthesia [8] or as a part of postoperative multimodal analgesia [9, 10]. Regional anesthesia in orthopedic surgeries provides numerous benefits, including improved postoperative pain control, reduced opioid consumption and opioid‐induced side effects, reduced postoperative nausea and vomiting, enhanced early postoperative mobilization, reduced risk of deep venous thrombosis, reduced morbidity and mortality, higher patient satisfaction, improved patient recovery, and reduced length of hospital stay [3, 11–13]. Nevertheless, these benefits of regional anesthesia are negated by the occurrence of rebound upon resolution of sensory block [14–16].

Rebound pain (RP) is an acute, burning, or dull‐aching type of pain that lasts around 2 h and occurs within 12–24 h after the resolution of regional anesthesia [17, 18]. It is defined as a “quantifiable difference in lowest pain scores when the block is working versus the highest acute pain encountered during 12 h after the effects of regional anesthesia resolve” [19, 20].

RP is a complex physiologic response that occurs due to an opposed nociceptive imputes and spontaneous C‐fiber activity after the effect of regional anesthesia wears off. In addition, RP might occur due to damage in peripheral nociceptors during regional anesthesia administration, increased injection pressure, myelin degeneration, and local inflammation at nerve endings [21, 22].

RP is a common complaint, specifically in orthopedic patients undergoing surgery under regional anesthesia. The occurrence of RP leads to significant consequences, including pronounced pain interference, increased consumption of opioids and other analgesics, higher patient dissatisfaction, delayed recovery, a greater risk of re‐admission, and increased hospital costs [17, 23–25].

Previous studies have explored the prevalence and determinants of RP; however, available evidence originates from high‐resource settings, where multimodal analgesia, adjuvant drugs, and advanced catheter‐based techniques are routinely practiced. The applicability of those findings to low‐resource environments is limited, as perioperative care pathways and anesthesia practices differ substantially. In resource‐limited settings, the limited availability of multimodal analgesic agents, adjuvant drugs for regional anesthesia, and continuous peripheral nerve block catheters, along with inadequate postoperative pain assessment and management protocols, may affect both the incidence and severity of RP. These factors underscore the importance of locally generated evidence to guide management within such contexts.

In Ethiopia, prior investigations have involved relatively small samples of orthopedic patients and have not comprehensively examined the predictors of RP severity. The present study was designed to address these gaps by determining the prevalence and predictors of RP following regional anesthesia in a large cohort of patients undergoing orthopedic surgery. Determining the prevalence and predictors of RP severity will help to identify high‐risk patients, individualize the pain management protocols, and enable clinicians to implement preventive measures, ultimately improving patient satisfaction and patient outcomes and reducing the burden of RP.

## 2. Methods and Materials

### 2.1. Study Design, Period, and Settings

An institution‐based cross‐sectional study was conducted at Debre Tabor Comprehensive Specialized Hospital (DTCSH) from April 1, 2024, to January 6, 2025. The hospital is located in the South Gondar zone, Debre Tabor town, Amhara Region, northwest Ethiopia. It is the only hospital in Debre Tabor town, serving a population of approximately 3 million people. The hospital has two operating rooms and a dedicated orthopedic ward containing 16 beds, which are specifically allocated for patients undergoing orthopedic surgeries.

### 2.2. Study Population

All consecutive adult patients (18 years and above) who underwent emergency or elective orthopedic surgical procedures under regional anesthesia alone or general anesthesia combined with regional anesthesia were included in our study. Patients who had a neuropsychiatric problem that impaired their communication, patients with unexpected intraoperative complications and who were admitted to the intensive care unit, and patients with primary regional anesthesia failure were excluded from our study.

### 2.3. Operational Definitions

RP: an acute transient burning type of postoperative pain that occurs within 12–24 h of the resolution of the sensory block of regional anesthesia [26, 27].

Rebound pain Score (RPS): quantifiable difference in lowest pain scores when the block is working versus the highest acute pain encountered during 12 h after the effect of regional anesthesia is resolved [10, 23, 28]. The patient is considered to have no RP if RPS is 0, mild if RPS is 1–3, moderate if RPS is 4–6, and severe if RPS is 7–10.

### 2.4. Study Variables

This study’s outcome variable was RP, classified as no, mild, moderate, and severe RP. Age, sex, body mass index (BMI), ASA physical status, presence of coexisting disease, severity of preoperative pain, premedication with intravenous dexamethasone, use of systemic analgesia, use of local anesthetics adjuvants, duration of surgery, and use of postoperative systemic analgesia were the independent variables of this study.

### 2.5. Sample Size Determination

The sample size of the study was determined by using a single population proportion formula.

(1) N=Z α22×p 1−Pd2,

where *N* = the required sample size, *Z* = the z‐score corresponding to the desired confidence level, *P* = proportion (prevalence of RP), and *d* = margin of error. By assuming a 95% confidence interval (CI) with a 5% margin of error and 61.7% prevalence of RP (23):

(2) N=1.962×0.617363.12364 10.617−0.052=≈.

### 2.6. Data Collection Procedure

The data were collected using a structured questionnaire that included sociodemographic, preoperative patient‐related, intraoperative, and postoperative variables related to RP. A direct interview with the participants and a review of their medical records were used to collect the data. The severity of preoperative pain, pain score before sensory block resolution, and pain score after sensory block resolution were assessed using an 11‐point numeric rating pain scale (NRS 0–10, 0 = *no pain* and 10 = *worst pain ever*).

### 2.7. Data Quality Control

A data collection supervisor and data collector were trained on how to collect data from patients and how to use the data collection tools. Regular supervision and follow‐up of data collection were conducted to ensure its completeness, accuracy, and clarity.

### 2.8. Data Management and Statistical Analysis Procedures

Once data collection was completed, the variables were coded and entered into EpiData software (Version 4.6) and then exported to Stata version 17 software for analysis. The Shapiro–Wilk normality test was used to evaluate the normal distribution of continuous variables. Categorical variables are presented as frequencies and percentages (%), while continuous variables are reported as medians with interquartile ranges (IQRs). The association between the outcome variable (severity of RP) and independent variables was examined using bivariable and multivariable ordinal logistic regression analyses. Independent variables with a *p* value of less than 0.20 at a 95% PCI in the bivariable analysis were included in the multivariable ordinal logistic regression analysis. Variables with a *p* value < 0.05 in multivariable analysis were considered significantly associated with the severity of RP. To determine the strength of the association between dependent and independent variables, crude odds ratio (COR) and adjusted odds ratio (AOR) with the corresponding 95% CI were calculated. The proportional odds (parallel line assumption) was checked and fulfilled with a *p* value of 0.24.

## 3. Result

### 3.1. Sociodemographic Characteristics of the Participants

A total of 364 participants who fulfilled the inclusion criteria were included in our study. The median (IQR) age of the participants was 46.5 (30–65) years, and 202 (55.5%) of them were under 60 years of age. Among the total participants, 273 (75%) were male, and 205 (56.3%) were in ASA physical status I. Of the total study participants, 57 (15.7%) had hypertension, 46 (12.6%) had diabetes mellitus (DM), 23 (6.6%) had bronchial asthma, 12 (3.3%) had epilepsy, and 7 (1.9%) had chronic obstructive pulmonary disease (COPD). Among the total participants, 142 (39%) had severe preoperative pain, 35 (9.6%) had moderate preoperative pain, and 90 (24.7%) had mild preoperative pain (Table 1).

**Table 1:** Sociodemographic and preoperative conditions of participants who underwent orthopedic surgery at DTCSH, 2025.

Variables	Variable category	Frequency (*n*)	Percentage (%)
Age	Below 60	202	55.5
	Above 60	162	44.5

Gender	Male	273	75.0
	Female	91	25.0

Coexisting disease	Hypertension	57	15.7
	DM	46	12.6
	Bronchial asthma	23	6.6
	Epilepsy	12	3.3
	COPD	7	1.9
	MI	6	1.7
	Chronic anemia	5	1.4

ASA physical status	I	205	56.3
	II	136	37.4
	III and above	23	6.3

Preoperative pain	No	97	26.7
	Mild	90	24.7
	Moderate	35	9.6
	Severe	142	39.0

Urgency of Surgery	Emergency	115	31.6
	Elective	249	68.4

*Note:* N = number.

Abbreviations: ASA = American Society of Anesthesiologists, COPD = chronic obstructive pulmonary disease, DM = diabetes mellitus, MI = myocardial ischemia.

### 3.2. Intraoperative and Postoperative Conditions of Participants

**Figure 1:**
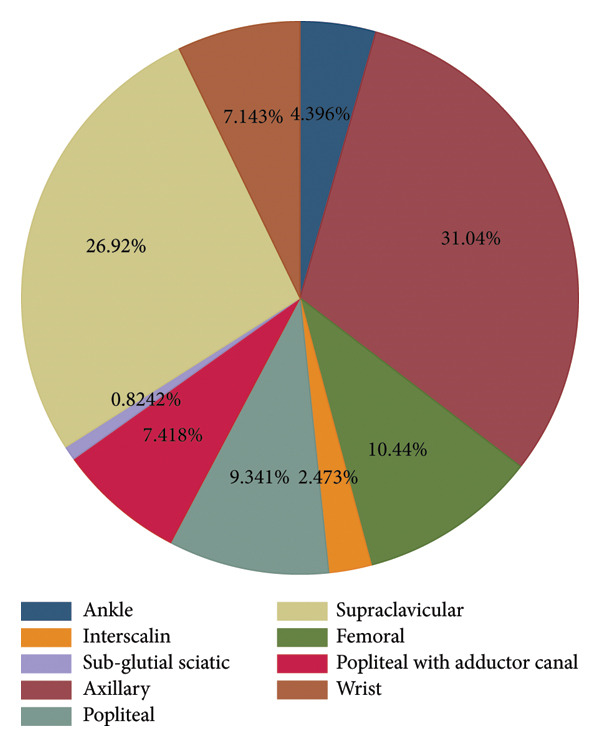
Types of regional anesthesia used in patients undergoing orthopedic surgery at DTCSH, 2025.

Of the total participants, 247 (67.9%) underwent upper extremity surgery and axillary nerve block was used in 113 (31%) participants (Figure 1). From the total participants, 112 (30.8%) underwent external fixation, 87 (23.9%) intramedullary nailing, 35 (9.6%) plating, 34 (9.3%) pinning, 28 (7.7%) nail removal, 23 (6.3%) sequestrectomy, 20 (5.5%) amputation, 15 (4.1%) incision and debridement, and 10 (2.7%) underwent pin removal. Among the total participants, preoperative and intraoperative systemic analgesia was used in 151 (41.5%) participants, and 166 (45.6) participants were premedicated with intravenous dexamethasone. Bupivacaine was used for nerve block in the majority (85.7%) of participants, and local anesthetic adjuvants were used in 281 (77.2%) participants (Table 2). The median (IQR) duration of motor and sensory block was 8 [7–11] hours and 10 [8–11] hours, respectively.

**Table 2:** Intraoperative and postoperative variables of participants who underwent orthopedic surgery at DTCSH, 2025.

Variable	Variable categories	Frequency (*n*)	Percentage (%)
Systemic analgesia	Yes	151	41.5
	No	213	58.5

Premedication with intravenous dexamethasone	Yes	166	45.6
	No	198	54.4

Surgical site	Upper limb	247	67.9
	Lower limb	117	32.1

Type of local anesthesia	Bupivacaine	312	85.7
	Lidocaine	52	14.3

Local anesthetic adjuvant	Yes	281	77.2
	No	83	22.8

Postoperative NSAID	Yes	232	63.7
	No	132	36.3

*Note:* N = number.

Abbreviation: NSAID = nonsteroidal anti‐inflammatory drug.

### 3.3. Prevalence of RP

In this study, the prevalence of RP after regional anesthesia was resolved was 70.9% (95% CI: 65.9–75.5). Among the total participants, 110 (30.2%) had mild RP, 70 (19.2%) had moderate RP, and 78 (21.4%) had severe RP.

### 3.4. Predictors of Severity of RP

In bivariable ordinal logistic regression analysis, the age of participants, preoperative pain, ASA physical status, premedication with intravenous dexamethasone, urgency of surgery, use of adjuvants, and postoperative NSAID use were significantly associated with the severity of RP, with a *p* value < 0.2. In multivariable ordinal logistic regression analysis, age less than 60 years, being ASA III and above, and having preoperative moderate and severe pain were significantly associated with increased odds of RP with a *p* value < 0.05. In contrast, premedication with intravenous dexamethasone and the use of local anesthetic adjuvants were significantly associated with decreased odds of RP.

Our study revealed that patients under 60 years of age had higher odds of experiencing higher RP (AOR: 2.34, 95% CI: 1.38–3.95, and *p*: 0.001) compared with those aged 60 and above. Moreover, patients with an ASA physical status of III or higher had nearly six times (AOR: 5.84, 95% CI: 2.17–15.69, and *p* < 0.001) higher odds of experiencing higher RP compared with those with an ASA I physical status. Patients with moderate preoperative pain had nearly three times (AOR: 2.86, 95% CI: 1.21–6.78, and *p* :  0.017) greater odds of higher RP compared with those who did not report preoperative pain. Likewise, patients with severe preoperative pain had increased odds of higher RP pain compared with those without preoperative pain (AOR: 7.48, 95% CI: 4.09–13.68, and *p* < 0.001).

Preoperative administration of intravenous dexamethasone reduced the risk of experiencing higher RP by 76% (AOR: 0.24, 95% CI: 0.14–0.43, *p* < 0.001). Moreover, using local anesthetic adjuvants decreased the odds of higher RP by 95% (AOR: 0.05, 95% CI: 0.02–0.11, *p* < 0.001) (Table 3).

**Table 3:** Bivariable and multivariable ordinal logistic regression analysis for factors associated with RP in patients who underwent orthopedic surgery at DTCSH, 2025.

Variable	Categories	COR (95% CI)	AOR (95% CI)	*p* value
Age	Below 60	6.25 (4.12–9.47)	2.34 (1.38–3.95)	**0.001**
	60 and above	Ref	Ref	

ASA physical status	III and above	6.39 (2.81–14.52)	5.84 (2.17–15.69)	**∼0.001**
	II	0.85 (0.57–1.26)	1.14 (0.68–1.93)	0.611
	I	Ref	Ref	

Preoperative pain	Severe	6.96 (4.17–11.59)	7.48 (4.09–13.68)	**∼0.001**
	Moderate	6.35 (2.97–13.59)	2.86 (1.21–6.78)	**0.017**
	Mild	0.33 (0.19–0.59)	0.59 (0.31–1.16)	0.129
	No	Ref	Ref	

Premedication with IV dexamethasone	Yes	0.14 (0.09–0.21)	0.24 (0.14–0.43)	**∼0.001**
	No	Ref	Ref	

Urgency of surgery	Emergency	3.62 (2.38–5.52)	0.93 (0.53–1.66)	0.828
	Elective	Ref	Ref	

Adjuvant used	Yes	0.02 (0.01–0.04)	0.05 (0.02–0.11)	**∼0.001**
	No	Ref	Ref	

Postoperative NSAID used	Yes	0.28 (0.18–0.41)	0.66 (0.41–1.08)	0.098
	No	Ref	Ref	

*Note:* *p* values < 0 .05 are considered statistically significant (bold).

Abbreviations: AOR = adjusted odds ratio, ASA = American Society of Anesthesiologists, CI = confidence interval, COR = crude odds ratio, IV = intravenous, NSAID = nonsteroidal anti‐inflammatory drug.

## 4. Discussion

This study aimed to determine the prevalence and predictors of the severity of RP after regional anesthesia in patients undergoing orthopedic surgery.

Our study revealed that the prevalence of RP after regional anesthesia was resolved was 70.9% (95% CI: 65.9–75.5). The finding of our study was relatively higher as compared with studies conducted in Ethiopia (61.7%) [23] and Canada (49.6%) [17]. The possible discrepancy for this may be due to differences in clinical setup and study population. Previous studies included patients who underwent orthopedic and nonorthopedic surgeries; in contrast, our study included patients who underwent orthopedic surgeries. As previous studies highlighted, surgical procedures involving bone are highly associated with RP [17, 23].

In our study, younger age (age less than 60 years) is significantly associated with experiencing higher RP severity. The finding is consistent with studies conducted in Ethiopia [23], Canada [17], Australia [29], and Denmark [8]. The possible explanation for this association is age‐related differences in pain nociception, perception, inflammatory response, and drug pharmacokinetics. Younger patients have lower pain threshold and express pain easily, whereas age‐related changes in nociceptors in older patients reduce the pain perception and occurrence of RP after the resolution of regional anesthesia. Additionally, younger patients often exhibit stronger inflammatory responses to surgical trauma, which may exacerbate peripheral sensitization and contribute to the occurrence of severe RP [30].

Additionally, our study demonstrated that patients having moderate and severe preoperative pain had higher odds of developing higher RP compared with those without preoperative pain. The possible explanation for this association is persistent nociceptive imputes from preexisting pain can trigger peripheral and central sensitization, which increases pain perception and results in hyperalgesia. These neuroadaptive processes contribute to increased nociceptive input and decreased pain threshold, ultimately leading to increased postoperative pain following the resolution of regional anesthesia [23, 31].

Our study also revealed that patients with higher ASA physical status are more likely to experience a higher severity of RP. From the scientific point of view, patients with higher ASA scores often present with different medical comorbidities, which can impact their physical, physiological, and mental well‐being. Such physical and psychological disturbances can result in depression and anxiety, which subsequently reduces pain modulation and increases pain perception [32].

Moreover, our study revealed that preoperative administration of intravenous dexamethasone reduced the risk of experiencing higher RP. The finding of our study is consistent with previous studies indicating that intravenous dexamethasone decreases both the incidence and severity of RP [17, 23, 33, 34]. The effectiveness of intravenous dexamethasone in reducing the incidence and severity of RP can be attributed to its prolonged sensory block and anti‐inflammatory effects. Intravenous administration of dexamethasone has been shown to prolong the duration of regional analgesia [35, 36], prolonging analgesic effects and reducing the incidence and severity of RP [34, 37, 38]. In addition, intravenous dexamethasone reduces the inflammatory response associated with surgical trauma. By inhibiting inflammatory mediators, intravenous dexamethasone decreases the excitability of nociceptors, diminishes pain signal transmission, and reduces sensitization of both peripheral and central pain pathways, ultimately leading to a decrease in the incidence and severity of RP [36, 39]. Besides its anti‐inflammatory actions, dexamethasone may attenuate postoperative pain via glucocorticoid receptor‐mediated modulation of nociceptive pathways, including suppression of spinal microglial activation and proinflammatory cytokine release, altered expression of pain‐related neuropeptides, and attenuation of central sensitization [40].

This study also demonstrated that the use of local anesthetics adjuvants significantly reduced the odds of developing higher RP. Adding local anesthetics adjuvants reduces the volume of local anesthetics required for nerve block, thereby reducing the extent of reversible local anesthetics‐induced neurotoxicity and ischemia. In addition, local anesthetics adjuvants prolong the duration of regional analgesia and enhance the quality and duration of analgesia. Collectively, these effects contribute to a marked decrease in the incidence and severity of RP [15, 19, 41].

This study addresses an important gap in understanding the severity and predictors of RP among orthopedic patients within a low‐resource Ethiopian healthcare context. By examining a larger, more diverse orthopedic cohort than previously reported, we identified specific predictors of RP, including younger age, ASA III status, preoperative pain severity, the use of intravenous dexamethasone, and local anesthetic adjuvants. These findings provide clinically relevant evidence for risk stratification and for optimizing perioperative pain management in resource‐constrained environments. The observed associations likely reflect underlying health system limitations, such as limited access to local anesthetic adjuvants, limited availability of multimodal oral analgesics, and the absence of standardized perioperative pain management protocols. Such constraints may intensify the transition from sensory blockade to unopposed pain once the effect of the local anesthetic wears off. Within this context, the use of intravenous dexamethasone and adjuvants and preoperative pain severity gain novel clinical significance. In high‐resource settings, availability of local anesthetic adjuvants, multimodal analgesic regimens and structured follow‐up often mitigate their impact, whereas in low‐resource systems these variables serve as practical, easily measurable indicators of indicators of patients at greater risk for severe RP. The limited postoperative follow‐up and restricted access to pain management strategies may further amplify these outcomes. This underscores the need for context‐specific pain management strategies suited to resource constraints.

This study has some limitations. First, its single‐center design may limit the generalizability of the findings to broader populations. Second, we did not assess the baseline preoperative anxiety score, the potential factor that could influence the occurrence of RP. Third, this study did not assess the possible association between the dose of local anesthetic adjuvant and the severity of RP. Fourth, due to the cross‐sectional design of our study and reliance on patient‐reported measures of RP severity, there might be a potential for selection bias, inaccuracies related to self‐reported data, and response bias.

## 5. Conclusion

Our study found a high incidence of RP following the resolution of regional anesthesia in patients undergoing orthopedic surgery. Younger age, an ASA physical status of III or above, and moderate to severe preoperative pain were significant predictors of higher RP severity. In contrast, administering intravenous dexamethasone preoperatively and using local anesthetic adjuvants were associated with lower odds of experiencing severe RP. To reduce the incidence and severity of RP, it is essential to effectively manage and reduce preoperative pain, incorporate local anesthetic adjuvants into nerve block solutions, utilize intravenous dexamethasone, and incorporate multimodal analgesia before the resolution of regional anesthesia. We recommend that future studies incorporate validated psychological assessment tools, such as the Generalized Anxiety Disorder‐7 (GAD‐7) and the Hospital Anxiety and Depression Scale (HADS), to evaluate anxiety and related psychological factors that may affect postoperative RP outcomes. In addition, further research is needed to examine the effect of varying doses of local anesthetic adjuvants on the incidence and severity of RP.

**Nomenclature**
AOR: Adjusted odds ratio
ASA: American Society of Anesthesiologists
CI: Confidence interval
COR: Crude odds ratio
NSAID: Nonsteroidal anti‐inflammatory drug
RP: Rebound pain

## Ethics Statement

Ethics approval was obtained from the research ethical review committee of the College of Health Sciences at Debre Tabor University with a reference number CHS 17/2024. The study was conducted in accordance with the Helsinki Declaration and followed the local legislation and institutional requirements. Informed consent was obtained from each study participant, and confidentiality was obtained by avoiding questions that contained identifiers.

## Consent

The authors have nothing to report.

## Disclosure

All authors read and approved the final manuscript.

## Conflicts of Interest

The authors declare no conflicts of interest.

## Author Contributions

Negesse Zurbachew Gobezie: conceptualization, data curation, methodology, formal analysis, software, writing–original draft, and writing–review and editing. Melaku Zewdu Yehualaw and Meraf Mehari Yitbarek: data curation, investigation, methodology, formal analysis, writing–original draft, and writing–review and editing. Getachew Mekete Deress, Kumlachew Geta Belete, Diriba Teshome, and Basazinew Chekol Demilew: investigation, supervision, software, visualization, and writing–review and editing. Temesgen Birlie Asmare, Habtie Bantider Wubet, Kaletsidk Dessalegn Mossie, and Begizew Yimenu Mekuriaw: methodology, data curation, validation, investigation, project administration, and writing–review and editing.

## Funding

There was no financial support from funding agencies (public, commercial, or nonprofit sectors) for research, authorship, or publication of the article.

## Supporting Information

The study follows the Strengthening the Reporting of Observational Studies in Epidemiology (STROBE) guideline to ensure clarity, transparency, and reproducibility in its design and reporting. The completed checklist used during manuscript preparation and submission is provided in the supporting file (STROBE‐checklist.docx).

## Supporting information


**Supporting Information** Additional supporting information can be found online in the Supporting Information section.

## Data Availability

The datasets used and analyzed during the study are available from the corresponding author upon a reasonable request.
